# Observations on Functional Disorders of the Spinal Cord, and Their Connexion with Hysteric, Nervous, and Other Diseases; Illustrated by Cases, Selected Chiefly from the Reports of the Pallas-Kenry and Currah Dispensaries

**Published:** 1830-02

**Authors:** Wm. Griffin, D. Griffin

**Affiliations:** Limerick.; Limerick.


THE LONDON
Medical and Physical Journal.

N° 372, VOL. LXIII.] FEBRUARY 1830. [N° 44, *New Series.*

For many fortunate discoveries in medicine, and for the detection of numerous errors, the world is indebted to the rapid circulation of Monthly Journals; and there never existed any work, to which the Faculty, in Europe and America, were under deeper obligations than to the *Medical and Physical Journal of London*, now forming a long but an invaluable series.—*Rush.*

### ORIGINAL PAPERS, AND CASES,

### OBTAINED FROM PUBLIC INSTITUTIONS AND OTHER AUTHENTIC SOURCES.

### FUNCTIONAL DISORDERS OF THE SPINAL CORD.

*Observations on Functional Disorders of the Spinal Cord, and their Connexion with Hysteric, Nervous, and other Diseases; illustrated by Cases, selected chiefly from the Reports of the Pallas-Kenry and Currah Dispensaries.*

By WM. GRIFFIN, M.D. and D. GRIFFIN, M.R.C.S. Limerick.

(Continued from page 25.)

SINCE it seems difficult to account for interruption of function, except by organic lesion or alteration, and the chief way in which such alteration makes itself known to us is by this interruption, it is not singular we should be very much puzzled when called upon to believe the ordinary actions of every organ of the body may be affected, either by preternatural increase or diminution, or total suspension, without the occurrence of any physical change. Hence it is that some pathologists deny altogether its possibility, looking upon the words *functional disease* as a sort of solecism, and asserting, with great truth, that, in a system so little admitting of minute examination as the nervous, most important changes may take place, which yet elude all observation. But, in speaking of functional disease, we believe, it is generally meant such affections as are not *traceable to altered structure*, and which admit of a perfect, and sometimes instantaneous, return to health. Whatever may be our reasoning, we have every day the indisputable fact before us, that people become suddenly attacked with symptoms resembling those resulting from organic changes, and yet as suddenly recover their original health, in a way which such changes would not seem readily to allow. These complaints may, perhaps, be said generally to depend on altered relations in the system, rather than on altered structure; and it is on this supposition that they have been so often attributed to vague and inscrutable sympathies. But, however mysterious they may be, our knowledge of their seat and character seems tolerably clear, derived as it is from the disturbance of function and other symptoms observed on particular changes of structure, and our experience of their usual course and termination. It is on this account chiefly, to give assurance of the connexion of those disorders dependent on irritation with particular parts of the system, as the spinal cord, we shall give, whenever we can, cases of organic disease of the same parts, presenting strong analogies of character.

We now proceed to point out more particularly the dependence of the many affections which we have mentioned, on irritation of a greater or less extent of the spinal medulla.

Although, for the sake of illustration, it is perhaps best to give the cases in the order which the affected portion would suggest, it is not intended to take up time with the simpler and less important ones. These, it is true, as being affections solely of the cervical, or the dorsal, or the lumbar portions, more strictly define the symptoms proper to each: but, independently of the necessity there will be to say something about them in a subsequent part of this paper, there are some reasons why they will not answer our purpose here. It seems necessary that a certain extent of the spinal cord should be in a state of irritation or disorder to produce any effect on the circulating system, or on the viscera. The simpler cases, therefore, depending on irritation of a point or limited portion of the medulla, are in general mere affections of one or two pair of the spinal nerves, sensitive or motory, mere neuralgia, and cannot furnish us with illustrations of the more important and complex irritative diseases, which it is proposed to reduce to some arrangement. It is only necessary to keep in view, that, in the cases cited as affections of the cervical medulla, the upper dorsal are sometimes implicated, and in those of the dorsal, sometimes the lower cervical, sometimes the upper lumbar. They must each, therefore, have some symptoms detailed not entirely proper to them, but which the intelligent reader will at once refer to their real origin.

*Irritation of the Cervical Portion of the Spinal Cord, inducing Affections of the Head, Neck, Chest, Stomach, and superior Extremities.*

Acute and chronic headach, browach, aching of the cheeks and face, may be mentioned first as among the most common symptoms of cervical irritation, both in the simple and complex cases. They are continually met with, as well as the subsequent ones of affections of the senses, in cases of organic disease of the cord, though usually in connexion with others of a more formidable nature. The following are taken almost indifferently from the case-book.

VIII. A young gentleman, aged twenty, complained of intense pain in the crown of the head and forehead, with excessive soreness of the scalp and feeling of general illness: is subject to attacks of the kind, and usually relieved by purgatives and lying down. There was great tenderness of the five upper cervical vertebræ, pressure on any of them occasioning the pain in the vertex and brow. Purgatives and rest were again successful in relieving him; the application of leeches, and a blister to the nape of the neck, to remove the tenderness, were then recommended. As long as this symptom remains, however effectual the relief, the complaint can only be considered as suspended.

IX. James O'Brien, aged fourteen years, applied at the dispensary, complaining of pain and soreness in the crown and forehead, especially on stooping, sometimes very distressing, and attended with deafness. There was tenderness of all the cervical vertebra; pressure on the first or second excited pain in the vertex and brow. Was ill one year. Recovered by the use of purgatives, and of blisters to the nape of the neck.

X. Ann Lynch, aged nineteen years, troubled with distressing headach, especially of the forehead, with sickness of stomach and thirst. Pulse ninety-five, tongue white, bowels confined; catamenia regular. Had been ill six days. Pressure on the first or second cervical, or behind the mastoid process, excited the pain severely at the brow. Was relieved by an emetic, followed by purgatives and a blister to the neck.

XI. Anne Day, aged thirty years, complained of constant headach, with soreness of the whole scalp; frequent faceach, affecting all the branches of the fifth pair. Has tenderness of the neck, and continued pain down the left arm and between the shoulders, at the left side of the spine.

Is unable to work; appetite impaired; general health bad. On examination, there was great tenderness of the cervical and upper dorsal vertebræ. This is a recent case, and yet under treatment.

XII. Mrs. M. aged forty years, a nurse, complained of headach, soreness of stomach, and soreness and pain of chest, with stiffness at the right side of the neck. This stiffness increased suddenly at times, seizing the muscles like cramp, and followed by hoarseness and dimness of sight. Was debilitated and in bad health. There was great tenderness on pressure at the middle cervical and seventh dorsal vertebræ. This patient was treated like the foregoing, but was slower in recovering.

The soreness of stomach was, in all probability, referrible to the tenderness at the seventh dorsal, and not, as it very frequently is, to irritation at the trunk of the par vagum. It is then usually accompanied by sickness and loss of appetite, and sometimes spasmodic attacks like cramp.

XIII. Mary O'Brien, aged forty years, ill three years, complains of pain in the head, particularly severe over the brows and at the temples, and occasionally confining her to bed for days. She is very weak and nervous; has no appetite, and is worse after eating. Is occasionally attacked with pain of stomach. On examination, there was found extreme tenderness of all the cervical vertebræ, pressure on any of them, or behind the mastoid process, exciting the pain severely at the brow and temples. There was also soreness of the seventh or eighth dorsal vertebra, pressure on which occasioned pain at the ensiform cartilage. In this case there was so much general debility, and so many points of the spine were affected for a length of time, that a rapid recovery was not to be anticipated. She did well after some weeks, by the strictest attention to the digestive organs, a course of tonics, and occasional small blisters to the spine.

XIV. Catherine Deely, aged thirty years, six weeks ill, complained of constant distressing headach, with pain in the stomach and nausea after eating. Bowels are in a natural state, but sometimes griped; catamenia regular. Pressure on the dentata excited the pain in the forehead, and on the ninth dorsal, at the stomach. Recovered under the use of mild aperients, with acids, and of counter-irritation.

These cases, which it would be tedious to multiply, serve to show the frequent dependence of painful affections of the head on spinal irritation. Perhaps it may be thought much that the mere fact of the distant pain being excited or aggravated by pressure on the cervical vertebræ, should establish such an inference; but, though this is in itself a very important circumstance, there are many others, hereafter to be considered, connected with the pathology of cerebral and spinal disease, which we think may more fully confirm it.

Among these, we cannot avoid remarking the pain and sickness of stomach so frequently mentioned as attendant on the headach. Indeed, the stomach is so seldom free, that the headach is usually looked upon as merely symptomatic of its disordered state; and so, no doubt, it often is; but much experience in these cases must convince us that the sickness and pain, and loss of appetite, are still oftener themselves symptoms, the result of irritation at the origin of the par vagum. It might, perhaps, be doubted whether disturbance of the stomach from any cause not acting immediately on itself, could ever exist without this irritation. We see that in inflammations, however extensive, of the arachnoid membrane, so long as it is confined to the convex surface of the brain, no effect is produced on it; but when the arachnoid of the base is attacked, vomiting is invariably one of the symptoms. Even the sympathies must have conductors, and, as it seems to be the general belief, these are to be found in the nervous system: we can only hesitate between the eighth pair and the sympathetic. There seem to be many reasons for believing that the latter is much less frequently the medium of communication between the head and stomach than the former.

Various affections of the senses, loss of sight, hemeralopia, loss of hearing, noises in the ears, vertigo, spectra or visions, delirium and insensibility, are severally effects of irritation at the cervical portion of the cord, and are sometimes accompanied by headach of a very intense nature. But they are still more frequently met with where the spinal affection is general. Blindness, vertigo, deafness, ringing in the ears, are affections that scarce need particular illustration, appearing as occasional symptoms in almost every severe case which we may have to relate. Hemeralopia is more unusual.

XV. John Hayes, aged fifteen years, complains that, as soon as night falls, he invariably becomes blind: he cannot see the furniture or people about the room, when they are perfectly visible to every one else. The candle or firelight appears a broad red haze, just distinguishable from darkness, but making nothing perceptible. He can perceive any dark object between him and the light, and no more. Has been affected in this way now about a fortnight, and had a similar complaint a year ago, which continued a good while. There is great tenderness evinced on pressing the second cervical vertebra. He perfectly recovered in less than forty-eight hours, by a small bleeding, an active calomel purgative, and a blister to the nape of the neck; and has since continued well.

The following is also a case in which vision was affected in an extraordinary manner.

XVI. A young gentleman, aged seventeen, is frequently attacked with violent headach and sickness of stomach, which symptoms were always ushered in by indistinctness of vision. His first warning of the fit is a sudden appearance of something misty and tremulous before his eyes; soon afterwards he can perceive only the vertical half of any object he looks at, and eventually the outlines fade away altogether into thick darkness. This almost total blindness continues generally for a very short period; the thick dark mist gradually clears off, and the forms of every thing around him are again distinctly observed. He is then instantly seized with intense headach, chiefly affecting the forehead, usually so dreadful in its nature, and accompanied by such distressing nausea or sickness, that he says he could scarce live if it lasted a second day. He commonly finds relief by lying down: the pain is thus more easily endured, and the paroxysm is shorter, terminating in four or five hours, when it might otherwise continue for twenty. Instead of pain, a deep lethargy sometimes supervenes on the affection of vision, during which he lies as in heavy slumber, but frightfully conscious of time passing, and of terrific sights and sounds thronging his imagination. He awakes out of this in a state of temporary delirium; does not know for some time where he is, or what has happened, and speaks incoherently. Even after the subsidence of the headach, although there is much less confusion of mind than after the lethargy, the memory is always very imperfect for some hours. He cannot recollect the words he wishes to make use of, but employs others wholly inapplicable in their stead; and of this mistake he is always conscious at the moment. To these attacks he has been subject for about two years, but in their intervals he has sometimes been affected in a very different way. He awakes suddenly out of his sleep at night in dreadful apprehension, for which he cannot account. There is a continued crowding and rushing of ideas through his mind. He feels as if every thing he did, and all that was done about him, passed over with a frightful and hurried rapidity. This at last wears away, and is generally, even from the first, more or less under the influence of his will; an effort to check the current of his ideas, and divert it into another direction, frequently proving successful.

On examination, there was found great tenderness of the second cervical and of the seventh or eighth dorsal vertebræ. When this last was slightly pressed upon, he felt a horrible sensation shoot through his whole frame. It was quite indescribable, and had nearly made him faint. He expressed the greatest apprehension at the thought of the pressure being repeated, and had a disagreeable feeling in his back for the entire day afterwards.

As this case fell accidentally within our notice, and was not at any time in our care, little of treatment can be given. He says the symptoms have been considered as the result of bile and a dyspeptic stomach, but that they do not seem to yield much to remedies, and still continue to recur as frequently as at their commencement. This is precisely what might be expected under general treatment. When once spinal disorder is induced, and has persisted for any length of time, it becomes, like chronic inflammation in the synovial membranes, altogether independent of its original cause, whatever that may be, and requires distinct attention. Attacks of the kind are often so troublesome and intractable as to be attributed to incurable organic disease; but although they may induce such mischief, if long neglected, they are usually, like symptomatic affections of the heart, the result of disturbed function alone. There can be scarce a doubt but, in the foregoing instance, the application of leeches, or cupping at the nape of the neck, repeated small blisters, followed by a stimulating liniment, to the whole of the spine, and strict attention to the general health, would have succeeded in effecting a cure.

There is much reason to think cases of amaurosis sometimes depend on irritation of the cervical portion of the medulla. The connexion of the 5th pair of nerves with the function of vision, which M. Magendie has so clearly ascertained, might readily suggest such a dependence. Indeed, this pair of nerves is so necessary to all the organs of sense, in the performance of their functions, that it does not seem extravagant to suppose these cases of slight insensibility, occurring in hysterical patients, may be sometimes caused by a temporary suspension or disturbance of its powers alone. They can neither hear, see, taste, or smell; they have only one faculty still connecting them with the surrounding world, general sensation; and over this the 5th pair has no influence.

The great discrepancy which prevails in the opinions of modern physiologists, both as to particular facts and the inferences to be drawn from them, is often exceedingly perplexing to the practitioner, who has seldom leisure or opportunity to repeat the experiments. Mr. C. Bell does not seem to think Magendie correct in his conclusions about the influence of the 5th pair over the nerves of the peculiar senses, and attributes, with much reason, the blindness, deafness, loss of smell and taste, which occurred in a case of extensive disease at its origin, to inflammation resulting from want of the common sensibility necessary to the protection of the different organs. It was so far an inappropriate illustration. But we cannot see how this is to explain the sudden loss of sight, or smell, or hearing, on division of the 5th, before inflammation can be said to exist, or the mischief which we may infer from the loss of common sensation could possibly occur. We can understand very well the necessary dependence of motion on sensation; but the dependence of the action of one sense on that of another is not capable of explanation, without admitting a new physiological law. Mr. Mayo's experiment of the division of the 5th pair within the cranium offers nothing conclusive on this subject, as the section of the nerve was imperfect. But it seems well worth remarking, that several eminent writers on diseases of the eye mention the frequent occurrence of amaurosis from wounds or injury of the supra or infra-orbitar branches of the 5th, and this without inflammation or the slightest observable change in the structure of the organ: in fact, Mr. Guthrie, who takes more particular notice of these affections, attributes them, not to any diseased action excited in the eye, but to sympathy; which is nothing more than supposing them to depend on some *unknown influence of the 5th* over the optic. In these cases, when alteration of structure is observable, Mr. Guthrie conceives there has been always an injury of the eye itself. There may perhaps be found, in the cases which we shall give, some pathological phenomena still more confirmatory of M. Magendie's inductions; but, if we were to search for illustrations, it would be difficult to find a more favorable one than is furnished by Mr. Bell himself, in the paper containing his comments on this subject: the case of Fred. Hill. Although it has been so recently before the public, we venture to restate it.

"Frederick Hill, ætat. ten, subject from childhood to a pain in his ear, was, twelve months ago, seized with obstinate pain of the left ear, which gave him no rest night or day. The pain extended to his head and face, and appeared sometimes to be *in the bones of his forehead and sockets of his eyes.* It then affected his teeth, and he had toothach *in every tooth in his upper jaw.* After this his left eye became affected, and *he lost his sight.* From this attack he recovered, as his mother says, by large bleedings, leeches, injections, shaving the head, and blisters. He had once or twice discharges of matter, preceded by pain, fever, and delirium, and followed at last by epileptic fits, on the recovery from which he was speechless. He was so irritable, that the slightest unexpected noise, even the striking of a clock, would bring on one of these fits; and having thrown himself down in a paroxysm of passion, about a week after his admission to the hospital, he became instantly deaf. His arm was afterwards observed to become useless: it hung by his side; he could move the fingers, but not the arm, from the middle of which to a little below the elbow was acutely painful when touched. The actions of respiration were perfect. When he smiled, there was no inequality in the action of the muscles of the face; he was said to make noise enough in laughing, and hollowed out when cupped, but there was no articulate sound. He understood every thing communicated by writing. When asked to speak, and the throat was grasped during the effort, there was no motion perceptible in the muscles of the tongue, yet he could masticate and swallow with ease; he could nearly touch the point of his nose with his tongue, or turn it down to the chin or sidewise. There seemed, nevertheless, an utter inability to the pronunciation of words; the consent of action between the chest, larynx, and mouth, seemed to be lost. The boy gradually recovered the use of his arm, and having left the hospital, suddenly recovered his hearing and power of speech at the same moment, some time after; a gush of matter taking place into the mouth at the time it occurred."

The explanation of this interesting case does not seem difficult. An abscess from disease of the temporal bone produced irritation at the base of the brain and medulla oblongata, "disturbing," as Mr. Bell justly expresses it, "the operations of the nerves, without altogether destroying their influence." The 5th pair became affected, and *there were pains in the forehead, in the teeth of the upper jaw, and blindness.* The seventh afterwards partook of the disturbance, or was affected through the 5th. There were finally irritability of mind, *delirium and epileptic fits, followed by speechlessness and paralysis of the left arm;* all of which are common results, as we shall afterwards show, of irritation at the upper part of the spinal column.

Mr. Bell does not say whether the blindness was sudden, but we may infer that it was. Had it been the result of inflammation, consequent to lost sensibility of the 5th, there must have been disorganization of the eye, as in Magendie's case, which it is evident there was not, as he perfectly recovered his sight; and had it been the result of disease at the origin of the optic nerve, the right eye should have suffered, and not the left, as the influence of these nerves is across, while that of the 5th is direct. The sudden recovery of hearing and of voice seems to make it yet more probable that all these symptoms might be fairly referred to irritation propagated from the internal ear to the base of the brain; in short, to functional derangement. And it is here necessary to call the attention of the reader to the remarkable similitude which this case presents to a train of symptoms occurring in the first one given in these papers. The lady, after suffering a length of time with an indisputable affection of the spinal column, in which *headach, aching of the forehead and facial branches of the 5th,* were prominent symptoms, was attacked suddenly with violent oppression, *blindness of the left eye, deafness of the left ear,* (omitted in the statement,) *paralysis of the left arm, and speechlessness.* None of these paralytic affections were perfect, except the last. The eye was slightly sensible to light, the ear to very loud sounds, and she could, like the boy Hill, move the fingers, but not the arm, but the voice was utterly gone. This seemed to depend on paralysis of the recurrent, and not of the lingual. The mind, the expiratory powers, and motions of the tongue and lips were perfect. She could express herself on paper, or make herself understood by the usual articulate motions, which it would appear the boy was unable to do.

The delirium mentioned as a symptom in the above case is a very common occurrence in spinal affections, whether chronic or acute. When unattended by feverishness, however wild or extravagant it may be, it is generally subdued without difficulty: sometimes, especially when it depends on uterine irritation, by one or two active purgatives. It is not rare to see a young girl, who is putting a whole house in an uproar, perfectly quieted by a dash of cold water and a dose of castor oil. We have no notes of cases precisely of this kind, but may offer the following, which seems to be of severer character than common, probably from long neglect.

XVII. Mary Enright, aged twenty-three years, is often seized with sudden blindness and giddiness, which in a short time pass off, but leave her with confused and disordered intellect. She describes herself as not knowing what she is doing, and saying all sorts of foolish things. This state lasts eight or ten days, after which she perfectly recovers her reason. The general health appears pretty good, and the catamenia regular. There is extreme tenderness of the second cervical, and of the seventh or eighth dorsal vertebræ: the pain in the last spot originated about nine months since, in consequence of a fall when carrying a can of water. This case, which bears a remarkable resemblance to the extraordinary one related above, was benefited by bleeding and purgatives; but is recent at the dispensary, and yet under treatment.

Many cases of ocular spectra, or illusions, seem also to belong to disorders of the cervical medulla; at least, if the spinal tenderness be admitted as any evidence of it. An instance occurred to us some years since, where a young girl was haunted by a spectral figure, which she described as standing by her bedside. She was frequently seized with fits of screaming, as she fancied it approached her, and kept her relatives in the greatest state of alarm and astonishment. A few active purgatives gave immediate and effectual relief. We shall only cite one case of a more recent date, presenting some singular features.

XVIII. Michael Nash, aged thirty-six, of a good constitution, but very intemperate habits, was for some days complaining of occasional pains in the stomach and arch of the colon, with costiveness, loss of appetite, and general nervous excitement. He had constant slight pain in the brow, with disturbance of vision, and extreme sensibility to noise, and all other impressions on the senses. His eyes were suffused; his tongue white; his pulse full, at about ninety; and he had flitting pain in his chest, with occasional oppressions and great anxiety. His chief distress, however, arose from visions, with which he was very continually troubled. Figures of persons, almost all of whom were wholly unknown to him, were frequently before him, sometimes so plain and distinct, that, although his reason assured him they were mere illusions, he could scarcely avoid believing they had an absolute existence. They were not always the same, nor always present, but went and came, renewing his anxiety and irritation of mind as often as they appeared. On examining the spine, tenderness was found at the three upper cervical vertebræ, pressure on any of them exciting the pain, with great suddenness, at the brow. The eighth, ninth, and tenth dorsal were excessively tender, the slightest pressure on any of them occasioning an exceedingly distressing sensation to shoot suddenly upward along the spine to the neck and brow, and downwards to the lower trunk and extremities. It was not pain, but a horrid feeling pervading the whole frame; and it was only by the greatest entreaty that he could be induced to permit a repetition of the examination. The sensibility had now so much increased, that a mere touch was sufficient to renew these distressing sensations. A pint of blood was taken from the arm. During the operation the visions returned: he said he saw three women standing behind the gentleman who was bleeding him. Being asked were they as large as life, he replied that "they were rather low," and pointed to the place where they stood. It was inquired "Had he ever seen them before?" No." "Were they speaking to each other?" "No." "What were they doing?" "They were usually minding their business, but sometimes stopped to watch him, and kept their eyes fixed on his for some moments." The sense of feeling was quite as much disturbed and illusive as that of sight; for in a few moments after he called out that he felt "one of them" thumping up against that part of the bed on which he lay; and presently again looked abruptly behind him, saying "that somebody had hit him two or three times on the back." All this was very different from the usual raving in mania, as he scarce felt the impression before he was himself aware of its illusion. In fact, the chief distress seemed to be from the imposing nature of the perception, when he knew it was impossible it could have had an object. "Get me rid of these sights and sounds," was his entreaty, "and get me some sleep, or I shall lose my senses."

Active purgatives were made use of after the bleeding, and a blister was applied over the ninth dorsal, which was of great service. He soon recovered under the continued use of gentle aperients, combined with bitters.

He had a recurrence of the attack some months after, in consequence of hard drinking; but though he complained more of the head, especially at the back of it, there was no material fulness or frequency of the pulse, or febrile irritation. He was relieved by purgatives and blistering, and was afterwards treated with camphor and other nervines.

Illusive affections of hearing, smell, and taste, are not perhaps so common as those of sight: they are yet found in frequent connexion with that acute sensitiveness of the whole nervous system which usually prevails in severe instances of spinal irritation. A lady, whose case we shall have hereafter to relate, was sometimes startled by distinct loud screams from one of her children, and could only be convinced of her illusion by sending to the hall, or the distant room from whence the sound seemed to come. This extreme sensitiveness appeared to be always connected with increased tenderness of the upper cervical portion of the cord.

We now come to speak of cases of much greater frequency and importance, those on which stupor, and sometimes coma, are induced. These have been usually attributed to affections of the brain, of which, indeed, they are frequently the result, but we shall have it in our power to show not by any means most frequently. We have before said it is impossible, in complaints rarely terminating fatally, to bring post-mortem or absolute evidence of their exact seat. We can only offer general reasoning. The extraordinary concurrence and resemblance of symptoms in those cases, we consider as functional disorders, with those recorded as having existed in the organic structure of the same parts. The physiological probability, the success of particular treatment, and the inference from strictly analogous cases: considerations of this kind, however individually unimportant, are strong and conclusive when corroborating one another; but we shall insist on them at more advantage towards the conclusion of these papers.

XIX. Margaret Fitzgerald, aged fourteen, a girl of sanguine habit, complained of slight headach and heaviness for some time, but did not seem otherwise unwell. Her countenance had usually an appearance of being flushed, and the eyes looked dull. One day, while reading, she fell suddenly upon the floor in a state of insensibility, but was not convulsed, and recovered very soon. She had three attacks of this kind within a short period of one another, before any medical treatment was instituted. Never had the catamenia.

On examination, great tenderness was discovered at the third cervical vertebra. Some blood was taken from the arm, and purgatives administered, which prevented the attack for a period of three months. On its recurrence, the same remedies were resorted to; but in eight or ten weeks there was a return of the fit. A blister to the tender vertebral bone was now ordered, and it was directed that it should be repeated in a few days, if the slightest soreness remained, or if at any time a recurrence of it was detected.

Nearly a year has now elapsed, and by a strict adherence to this plan she has never since had a fit. There have more than once been strong indications of its approach, in the appearance of the countenance, and a return of the tenderness in the neck; but purgatives and blisters always prevented the attack.

XX. Mary Caugney, aged eleven years, was for some days accustomed to fall down in a fit, in which she lay for some time, with flushed face, speechless, and almost insensible, generally from one to four hours. They came on sometimes two or three times a day. The tongue was white; the pulse natural, at ninety; she occasionally felt pain in the bowels and head, sometimes a little preceding the fit. The pain in the head was instantly brought on by pressure on the cervical vertebræ, particularly the second and third. The sixth, seventh, eighth, and ninth dorsal are also tender to the touch; pressure on the third, fourth, or fifth dorsal, sent a shooting pain up the neck and over the brow, which was felt as if passing within the canal of the medulla spinalis.

In this instance purgatives were made use of for seven or eight days, with little effect. The fits became less frequent, but still continued to recur. Six ounces of blood were then taken from the arm, and a blister ordered to the nape of the neck. So much relief was derived from the bleeding, that the girl neglected to apply the blister, and continued well and free from the fits, until the beginning of June, (about six weeks afterwards,) when they again recurred with precisely similar symptoms. The abstraction of blood being now repeated, and followed by the application of a blister, the attack was arrested as before, and has not since returned.

These cases are of exceeding interest, if only as illustrations of apparently formidable diseases yielding so instantaneously to the treatment. We may venture to infer from them, as well as from numerous others, how much more frequently even affections of the senses are the result of disturbed function than of organic mischief. These fits are frequently, as in the latter case, interrupted or cured by abstraction of blood. Whether this acts by relieving congestion in the spinal vessels, as many have supposed, or by quieting nervous irritation, we must not now stay to inquire. The attack is too frequently mistaken, especially in children, for a serous affection of the head; but is almost invariably at first symptomatic of some distant irritation, as of dentition, disordered stomach or bowels, or of a peculiar state of the uterine system; to which probably it was to be attributed in one of the foregoing instances. It is continually met with in all those spinal or hysteric cases in which the cord is affected to any extent; and though usually, and perhaps necessarily, connected with a deranged state of the cervical medulla, it seems sometimes to occur when the lower portions are more severely engaged; even so low as the middle lumbar. It is often attended by pallor and universal coldness of the surface, with depressed circulation, and is then mistaken for syncope, which in fact it closely resembles; but much more frequently it is accompanied by flushing of the cheeks and brow, with throbbing of the carotid and temporal arteries, and burning heat in the hands and forehead. We have never seen it occasion stertorous breathing, but, when it terminates fatally, it may possibly do so, running into perfect apoplexy. It may, however, end in death without exhibiting any such symptoms, as it sometimes seems to do in young children; in women with spinal affections subsequent to delivery; and we may perhaps say in those singular instances in which typhoid patients die suddenly in their convalescence.

Cases of this description occur occasionally in the slightest as well as in the most severe instances of spinal irritation, and are not commonly dangerous in either, unless altogether neglected or mistreated. We have seen a patient remain ten or twelve days in a state of insensibility, only differing from apoplexy in the quiet, almost imperceptible, breathing, and the greater mobility of the iris; and from syncope, in the distinct, full pulse, the temperature of the skin, and the natural expression of countenance. This is very like what has been called trance, but not bearing so perfect a resemblance to death as that state is sometimes said to do. It is not uncommon, however, to see a child so affected during dentition, that it is with difficulty distinguished from one that is recently dead. The subject of fits affecting infants is one of extreme interest, if only from their frequent occurrence, but still more so from the great and sudden danger with which they are often attended. This fortunately depends not so much on the violence of the exciting cause as upon the extraordinary sensibility and irritability of fibre which exist at that tender age. About their proximate cause there has been some discussion, and very lately a paper has appeared in one of our periodicals, from an American physician, who attributes them in all instances to spasm affecting the intestines. This spasm, he conceives, may be induced by disordered stomach and bowels, the common result of the irritation and feverishness of dentition, or of flatulence or acidity arising from improper food. The fit of insensibility or convulsion is thus invariably regarded as a sympathetic effect of the spasm. No doubt, it would be supposed, could be entertained that worms, flatulence, and acidity, by irritating the intestinal tube, may, whether exciting spasm or not, induce the sympathetic action on the sensorium; but, in the cases originating in dentition, which are by much the most numerous, it will appear obvious that the fit is excited in a more direct way, the irritation at the extremity of the maxillary branch of the 5th pair inducing a peculiar affection at the base of the encephalon or medulla oblongata, which in its turn occasions either the disorder, and perhaps spasm of the stomach, or the state of insensibility or convulsion, or both, as we have seen it continually does in severe instances of spinal irritation in adults. It is necessary to dwell a little on this point, as, if our opinion is correct, it may influence materially the views of treatment. No fact seems clearer to us than that those cases in which the sensorium is affected in dentition, through the medium of the bowels, are by no means the most frequent; that the disturbance of the bowel is itself commonly an effect of spinal irritation; and that even where it is the medium by which the inflamed gums act, it is by no mysterious sympathy between them and the brain, but usually by bringing on that peculiar state of the medullary cord, of which so much has been already said. It is unsatisfactory that this state cannot be asertained by examination of the spine, as in grown persons: children are apt to cry at very slight pressure, when perhaps there is no soreness at all; and yet it does not appear to us that an attentive observer would often be deceived in the expression of the countenance. The sharp, quick wincing of the features, when acute pain is felt, is very different from their slow gathering up in mere peevishness. The following case seems strikingly to support the foregoing opinions.

XXI. An infant of six or eight months old, apparently in good health, but suffering at times from teething, became restless in the nurse's arms, and was seized with sudden paleness. He had slight quivering and blueness about the lips, the eyes turned upwards, and he lay back still and insensible, as in syncope or death. He was recovered by the warm bath; his gums were freely lanced, and the bowels were strictly attended to. He had, however, a recurrence of the fit every six or seven days, sometimes to a prolonged and alarming degree. The state of the spine now excited attention, and, as the child seemed to evince strong indications of pain on slight pressure at the upper cervical, a blister was applied there. The fit did not return for a fortnight, and then in a slighter degree than usual. The blister was consequently repeated, and another laid behind each ear, as soon as it healed. After this three weeks elapsed before there was a recurrence of the attack. It was at length determined to keep some part of the back of the neck constantly in a state of irritation; and, as long as this plan was adhered to, the child continued perfectly well. All apprehension on the subject ceased after some weeks, and the soreness behind the ears having been unadvisedly allowed to heal up, the little patient was once more seized with the fit, while asleep in the cradle, to a more violent degree than ever, and recurring two or three times in the course of one night: recourse was instantly had to the remedy which had already proved so successful, and, from this time until the irritation of dentition had entirely subsided, a slight discharge was constantly kept up, either behind the ears or at the back of the neck. The child never had a return of the attack afterwards.

However deeply impressed with a conviction that fallacies in medical reasoning often originate in inferences drawn from single cases, we cannot but observe, that here the treatment originated in conclusive deductions from strictly analogous affections, and that no remedy directed simply either to the bowels or the teeth, or the nervous system generally, was of any avail. It was the strong similitude observed between those attacks in infants, and the fits of insensibility and convulsions occurring in girls of hysterical habits, known to depend on spinal irritation, that first led to our attributing them to a like cause. Women, possessing greater sensibility of the nervous system than men, are much more liable to violent affections of it from very trivial causes, whether mental or corporeal, which accounts for the fact that functional disorders of the spinal cord (nervous diseases) are esteemed almost peculiar to them. Infants have, as has been already observed, the same exquisite texture and susceptibility to slight impressions, and we should therefore naturally expect would be subject to the same complaints.

Fits of insensibility are frequently brought on by slight pressure at the affected vertebra; a convincing proof of their dependence on the state of the medulla. We shall have to state cases, when speaking of general irritation of the cord, in which patients tumbled suddenly forward, perfectly insensible, on pressing at a particular point of the spine. Fits of syncope are occasionally brought on in the same way, as might have probably occurred in Case XVI. of the young gentleman whose vision was so singularly affected, had the pressure been continued or repeated. Indeed, as we have before mentioned, it is sometimes extremely difficult to distinguish between fits of insensibility in which the heart and arterial system are only secondarily affected, and fits of syncope in which they are directly depressed. Of these last we shall next proceed to speak.

